# Impact of Exams on Diet, Physical Activity, and Body Composition in University Students

**DOI:** 10.3390/nu17111929

**Published:** 2025-06-04

**Authors:** Natalia Mudarra-García, Marina Pérez-Mudarra, Ismael Ortuño-Soriano, Raquel Badía-Iborra, María Jesús Vicente-Galán, Ignacio Zaragoza-García, Fernando Roque-Rojas, Francisco Javier García-Sánchez

**Affiliations:** 1 Research Nursing Area, Hospital Universitario Ramón y Cajal, Instituto Ramón y Cajal de Investigación Sanitaria (IRYCIS), 28034 Madrid, Spain; nmudarra@enf.ucm.es; 2 Nursing Department, Faculty of Nurse, Phisiotherapy and Podology, University Complutense of Madrid, 28040 Madrid, Spain; iortunos@ucm.es (I.O.-S.); mvicen14@ucm.es (M.J.V.-G.); izaragozac@ucm.es (I.Z.-G.); 3 Department of Teaching, Alfonso Moreno Institute, 28690 Brunete, Spain; marina.perez.mudarra@gmail.com (M.P.-M.); raquel.badia@educa.madrid.org (R.B.-I.); 4 Instituto de Investigación Sanitaria Hospital Clínico de San Carlos (IdISSC), 28040 Madrid, Spain; 5 Instituto de Investigación Sanitaria Hospital Universitario Fundación Alcorcón (iHUFA), 28922 Alcorcón, Spain; 6 Instituto de Investigación Hospital 12 de Octubre (IMAS12), 28041 Madrid, Spain; 7 Surgical Prehabilitation Unit, Hospital Universitario Infanta Cristina, Instituto de Investigación Sanitaria Hospital Puerta de Hierro Segovia Arana (IDIPHISA), 28981 Madrid, Spain; fernando.roque@salud.madrid.org; 8 Medical Department, Faculty of Medicine, University Complutense of Madrid, 28040 Madrid, Spain

**Keywords:** body composition, dietary habits, physical activity, stress, bioimpedance, students, university students, exams

## Abstract

Background: Bad dietary habits and sedentary lifestyles alter body composition, increasing disease risk. Methods: We conducted a prospective, comparative, longitudinal observational study among nursing students from Complutense University of Madrid. Data were collected in two periods: before exams (January 2024) and during exams (May 2024). Body composition was assessed via bioimpedance (Beurer BF 1000), dietary habits through the modified Kidmed survey, and physical activity using the IPAQ. Results: During exam preparation, fat mass significantly increased (25.43% to 28.79%, *p* = 0.016), muscle mass significantly decreased (39.70% to 36.20%, *p* < 0.001), and visceral fat rose notably (2.34 to 3.52, *p* < 0.001). Students exhibiting poor dietary quality increased (54.2% to 80.0%, *p* < 0.001), vigorous physical activity dramatically decreased (84.7% to 11.1%, *p* < 0.001), and sedentary time increased significantly (408.24 to 543.61 min/day, *p* < 0.001). Conclusions: Our findings suggest dietary deterioration and reduced physical activity during exams adversely affect students’ body composition.

## 1. Introduction

University students are frequently exposed to high levels of academic stress, particularly during exam periods. This stress can lead to poor lifestyle habits such as unhealthy eating, reduced physical activity, and increased sedentary behavior [1,2]. These changes, in turn, may negatively affect students’ physical and mental health [3].

Maintaining healthy dietary patterns and engaging in regular physical activity are essential to preserving body composition and preventing chronic conditions such as obesity, cardiovascular disease, and metabolic disorders [4]. However, university students often exhibit poor adherence to healthy habits, especially during periods of academic overload [5,6]. The exam period, in particular, is associated with increased psychological distress, time constraints, and disrupted routines, all of which may compromise self-care behaviors [7].

Changes in diet, including increased consumption of processed foods, skipped meals, and irregular eating patterns, are commonly observed among students under stress. Simultaneously, levels of physical activity tend to decrease, while sedentary time rises, further aggravating the potential impact on health and well-being [8,9,10].

Previous studies have assessed the effects of academic stress on perceived health, psychological states, and academic performance. However, few have focused on the direct impact of exam periods on objectively measured changes in body composition [11]. The originality of the present study lies in the integration of validated tools to assess diet, physical activity, and body composition before and during exams, providing a comprehensive view of how academic stress affects nursing students’ health.

This study aims to evaluate whether the period of exam preparation and associated stress results in significant alterations in body composition and lifestyle habits among university students.

## 2. Materials and Methods

### 2.1. Study Design and Population

This study was designed as a prospective, observational, comparative, and longitudinal investigation. The target population consisted of second-year undergraduate nursing students enrolled at the University Complutense of Madrid.

This study was approved by the Ethics and Research Committee of Puerta de Hierro University Hospital (ACT 244/23). Access to the study files was password-protected and restricted to the responsible researchers in accordance with current data protection regulations (BOE-A-2018, Regulation 2016). The study was also approved and registered at ClinicalTrials.gov with the identifier NCT06717620.

The required sample size was estimated using G*Power 3.1.9.7 for paired-sample t-tests, considering a medium effect size (d = 0.5), alpha = 0.05, and power = 0.8, resulting in a minimum of 34 participants. Our final sample (n = 72) exceeded this requirement.

Of the initial 142 students recruited, 72 completed both evaluation phases. The main reasons for dropout included scheduling conflicts, lack of availability on the second evaluation day, and incomplete data in either the body composition assessment or questionnaire.

#### 2.1.1. Inclusion Criteria

- Age ≥18 years;
- Current enrollment in the second year of the nursing program;
- Willingness to participate in both evaluation periods.

#### 2.1.2. Exclusion Criteria

- The presence of any physical limitation that prevented bioimpedance measurement;
- Ongoing use of pharmacological treatments that could influence body composition;
- The inability to complete the second assessment (e.g., absence on the exam day).

### 2.2. Intervention

Participants were assessed at two time points: (1) prior to the start of the exam preparation period (January 2024), and (2) during the exam period, immediately following completion of a major exam (May 2024).

**Figure 1:**
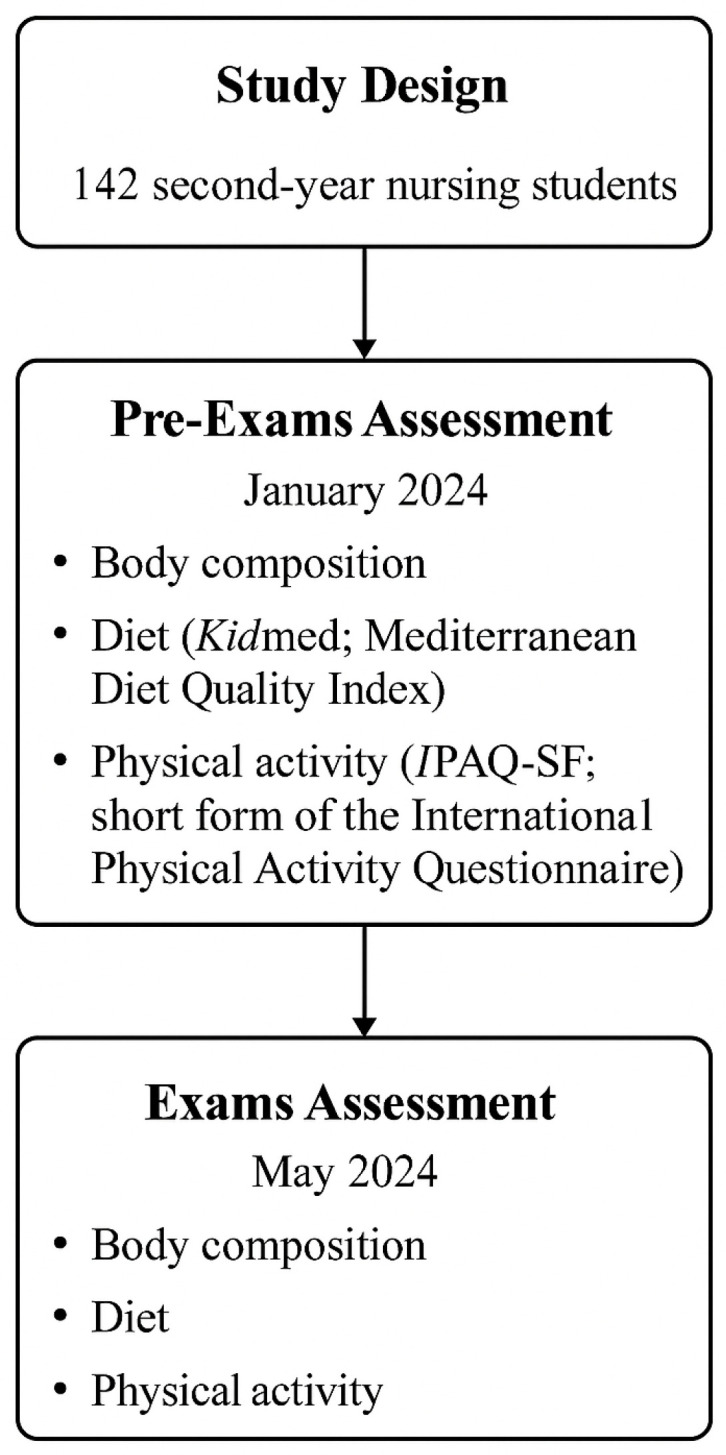
Flowchart of the study design. The study involved 142 second-year nursing students who underwent two assessments: one before the exam period (January 2024) and another during the exam period (May 2024). Each assessment included measurements of body composition (via bioimpedance), dietary habits (Kidmed questionnaire), and physical activity levels (IPAQ-SF).

At each time point, the following data were collected:

- Anthropometric and body composition measurements: Body weight (kg), height (cm), fat mass (%), muscle mass (kg), and visceral fat (score) were assessed using a validated bioimpedance device (Beurer BF 1000). Participants were instructed to remove footwear and metallic items and to follow standard pre-measurement conditions (e.g., fasting state, avoidance of intense physical activity 12 h prior to measurement).
- Dietary habits: Evaluated using a modified version of the Kidmed questionnaire (Table A1), which assesses adherence to the Mediterranean diet. The questionnaire includes positive and negative dietary items, with a scoring system that classifies diet quality into low (≥3 points), medium (4–7 points), or optimal (≥8 points).
- Physical activity: Measured using the short form of the International Physical Activity Questionnaire (IPAQ-SF). Participants reported the frequency and duration of vigorous, moderate, and walking activities over the past 7 days. Total physical activity was calculated in MET-minutes/week and categorized as low, moderate, or high following standard IPAQ classification criteria (Figure 1).
- Additional data on lifestyle behaviors (e.g., caffeine consumption, tobacco use, intake of cola or energy drinks) were also gathered via a structured survey.
- The effect of the menstrual cycle on bioimpedance measurements was not controlled for. This is acknowledged as a limitation of the study.

### 2.3. Statistical Analysis

Descriptive statistics were used to summarize sociodemographic and clinical characteristics. Continuous variables were expressed as means and standard deviations, and categorical variables as absolute frequencies and percentages. Normality was assessed using the Kolmogorov–Smirnov test to determine whether parametric or non-parametric tests were appropriate. Paired-sample t-tests were used for normally distributed continuous variables, while Wilcoxon signed-rank tests were applied to non-normally distributed data. Chi-square tests assessed differences in categorical variables. The statistical significance threshold was set at *p* < 0.05. All analyses were performed using IBM SPSS Statistics for Windows, Version 29.0 (IBM Corp., Armonk, NY, USA).

## 3. Results

### 3.1. General Characteristics of the Population

In the first phase of data collection, conducted in January 2024 (non-exam period), a total of 142 students were initially recruited. After applying inclusion criteria and completing both phases, 72 students were included in the final analysis. Most participants were female (78.9%), with a mean age of 22.54 years. The mean body weight was 63.32 kg, with a mean BMI of 23.04. Regarding body composition, 42.3% of the students were categorized as having very high body fat, and 50.7% were within the normal muscle mass range.

Diet quality was classified as medium in 48.6% of participants and optimal in another 48.6%. Physical activity was high in 80.3% of the sample during this period (Table 1).

### 3.2. Comparative Results Between Pre-Exam and Exam Periods

In the second phase of the study, conducted in May 2024 (on the day of the nutrition exam), 72 students completed both data collection stages and were included in the comparative analysis. Of these, 79.2% were female, with a mean age of 21.94 years (Table 2).

#### 3.2.1. Significant Changes Between the Two Phases

**Figure 2:**
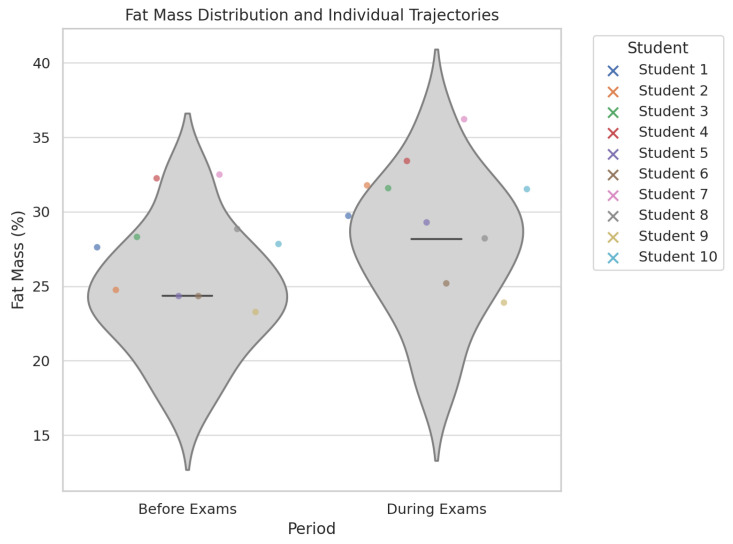
This figure illustrates individual trajectories in fat mass for a random subset of 10 participants, for visualization purposes. Violin and box plot of fat mass distribution before and during exams, including individual trajectories for a subset of 10 students. The figure highlights both the shift in distribution and the within-subject variability associated with academic stress.

- Fat mass increased from a mean of 25.43% pre-exams to 28.79% during the exam period (*p* = 0.016).
- Muscle mass decreased from 39.70 kg to 36.20 kg (*p* < 0.001).
- Visceral fat rose from a mean of 2.34 to 3.52 (*p* < 0.001).
- The percentage of students with very high body fat increased from 38.9% to 63.9%, and those with high muscle mass decreased from 36.1% to 8.3% (Figure 2).

#### 3.2.2. Significant Changes Observed in Dietary Habits

**Figure 3:**
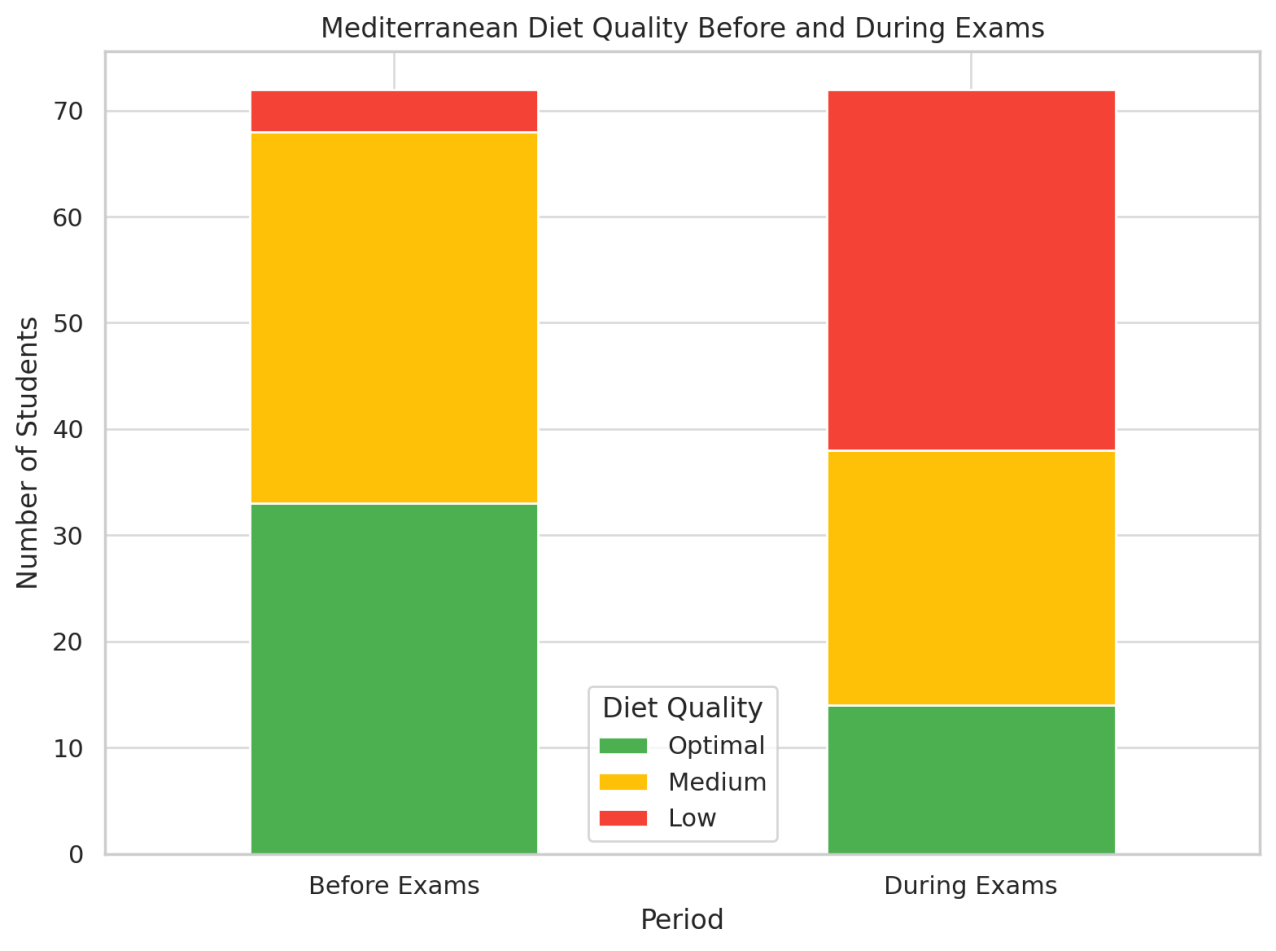
Stacked bar chart showing the distribution of Mediterranean diet quality among students before and during the exam period. The figure illustrates a marked decline in diet quality during exams, with a substantial increase in the number of students classified as having low adherence to the Mediterranean diet.

- Students consuming a second piece of fruit daily decreased from 52.8% to 33.3% (*p* = 0.018).
- Fast food consumption increased from 48.6% to 52.8% (*p* = 0.024).
- The proportion of students skipping breakfast rose from 13.9% to 51.4% (*p* < 0.001).
- Consumption of pastries for breakfast increased from 23.6% to 45.8% (*p* = 0.005).
- Daily candy consumption increased from 6.9% to 29.2% (*p* = 0.001).
- Optimal diet quality decreased from 45.8% to 20.0% (*p* < 0.001), with a drop in mean Kidmed score from 7.55 to 5.45 points (*p* < 0.001) (Figure 3).

#### 3.2.3. Significant Changes Observed y Physical Activity

**Figure 4:**
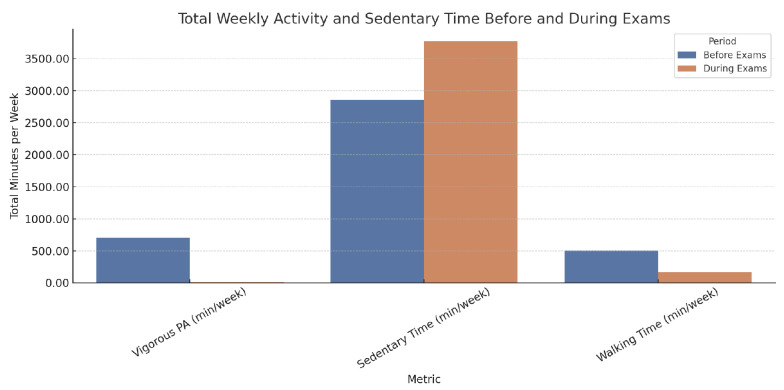
Total weekly minutes of vigorous physical activity, sedentary time, and walking before and during the exam period. The figure illustrates a significant decline in physical activity and walking, alongside a marked increase in sedentary behavior during exams. Data are based on calculated totals from self-reported frequency and duration.

- Days of vigorous physical activity decreased from 3.10 to 0.72 days/week (*p* < 0.001).
- Vigorous activity time dropped from 229.02 to 30.69 min/week (*p* < 0.001).
- The proportion of students with low physical activity rose from 4.2% to 59.7% (*p* < 0.001), while those with high physical activity dropped from 84.7% to 11.1%.
- Mean sitting time increased from 408.24 to 543.61 min/day (*p* < 0.001) (Figure 4).

### 3.3. Impact of Diet Quality on Body Composition

**Figure 5:**
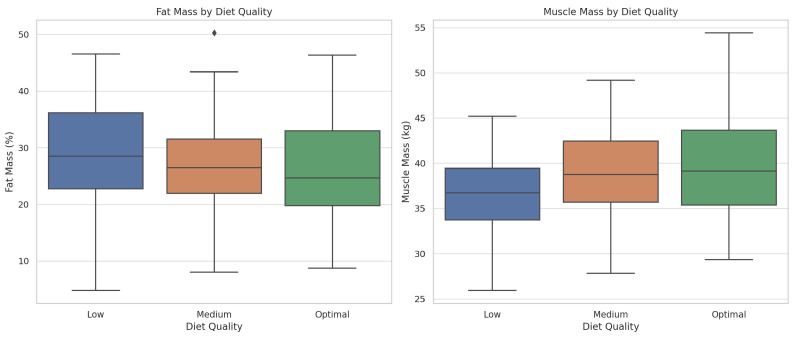
Distribution of fat mass and muscle mass according to diet quality. The boxplots illustrate that students with lower diet quality (as classified by the modified Kidmed score) tend to have higher fat mass and lower muscle mass, reinforcing the association between nutritional habits and body composition.

Students with poor diet quality showed worse body composition parameters, with higher fat mass (30.10%), higher visceral fat (4.14), and lower muscle mass (35.16 kg) compared to those with optimal dietary habits (Table 3; Figure 5).

### 3.4. Impact of Physical Activity on Body Composition

Similarly, students with low levels of physical activity had higher fat mass (30.31%), higher visceral fat (3.90), and lower muscle mass (35.25 kg) compared to their more active peers (Table 4).

**Figure 6:**
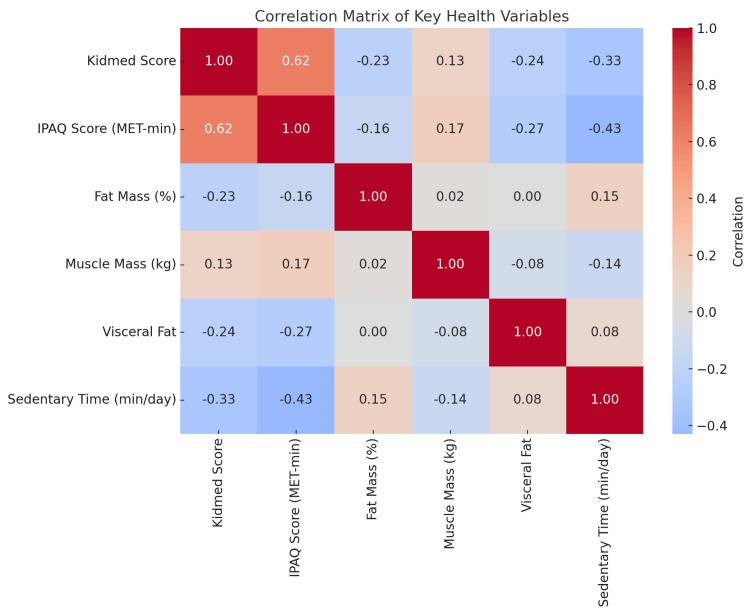
Correlation matrix of key variables related to lifestyle and body composition. The heatmap displays Pearson correlation coefficients between the Kidmed score, IPAQ physical activity score (MET-min/week), fat mass, muscle mass, visceral fat, and sedentary time. Strong inverse correlations are observed between Kidmed and IPAQ scores and fat-related measures, highlighting the impact of healthy habits on body composition.

Figure 6 displays the correlation matrix of the main study variables. Higher adherence to the Mediterranean diet, as assessed by the Kidmed score, was positively correlated with physical activity levels (r = 0.62) and negatively correlated with sedentary time (r = −0.33), fat mass (r = −0.23), and visceral fat (r = −0.24). Similarly, IPAQ scores showed a strong negative association with sedentary behavior (r = −0.43) and moderate negative associations with fat mass and visceral fat. Notably, sedentary time was inversely correlated with both dietary quality and physical activity, reinforcing its role as a negative determinant of body composition.

## 4. Discussion

The most relevant findings of this study clearly demonstrate the relationship between dietary habits, physical activity, and body composition among university students. The results show that students with poor diet quality and low physical activity levels exhibited increased fat mass, reduced muscle mass, and elevated visceral fat. In particular, the increase in visceral fat observed in students with unhealthy lifestyle habits is concerning, as sustained high levels are associated with a higher risk of developing metabolic disorders such as T2D and cardiovascular diseases, including hypertension and dyslipidemia. These results support the findings proposed by Maza and colleagues [3].

Furthermore, the instrument used to assess physical activity in this study, the short form of the International Physical Activity Questionnaire (IPAQ-SF), has been validated in international research and is considered suitable for population-level surveillance. As highlighted by Mantilla Toloza and Gómez-Conesa (2007) [12], the short version is especially recommended for regional and national prevalence studies due to its adequate reliability (r = 0.76; 95% CI: 0.73–0.77) and its practical application across diverse populations. Although the IPAQ-SF does not provide as detailed information as its longer counterpart, it effectively captures essential domains of physical activity—such as walking, moderate and vigorous activity, and sedentary time—which are particularly relevant during periods of lifestyle disruption, such as university exam sessions. Therefore, its inclusion in this study was both methodologically justified and operationally feasible given the academic setting and time constraints of the participants [12].

Another important observation is that body weight and BMI did not differ significantly between the two assessment periods. This can be explained by the balance between increased fat mass and decreased muscle mass [13]. Although body weight remained relatively stable, the underlying body composition worsened, emphasizing that weight and BMI alone are not sufficient indicators of health status. Assessing fat and muscle mass provides a more accurate picture of metabolic and nutritional health [11].

The previous literature supports the notion that academic stress during examination periods [14] and negatively impacts students’ lifestyles [15,16]. Increased academic demands often lead to disrupted routines, poorer eating habits [5], and a reduction in physical activity levels [17]. Students may skip meals, consume ultra-processed foods [7], or neglect regular exercise due to time constraints or psychological stress, which, over time, can adversely affect body composition [18] and overall health [19].

These findings highlight the need to include stress management strategies within academic environments, such as mindfulness techniques, relaxation exercises, and structured study plans that allow time for self-care. Moreover, educational initiatives focused on promoting healthy eating and regular physical activity should be integrated into university curricula to raise awareness among students about the long-term consequences of unhealthy behaviors.

Despite limitations such as the dropout rate between the two assessment points, the study offers evidence of the detrimental effects of stress-related lifestyle changes on body composition. Although the internal validity of this study is limited due to the homogeneous sample (students from a single university), the external validity is notable, as similar behaviors and stress patterns are likely to affect student populations in other academic settings.

Ultimately, the negative impact of poor habits during exam preparation affects a large portion of the student population. Addressing this issue requires institutional efforts to promote lifestyle interventions aimed at preserving both physical and mental health during high-stress academic periods.

Based on our findings, we recommend that universities consider implementing preventive strategies during exam periods, such as brief nutritional counseling, stress management workshops, and promotion of short, adaptable physical activity routines to preserve students’ physical and mental well-being.

## 5. Conclusions

**Figure 7:**
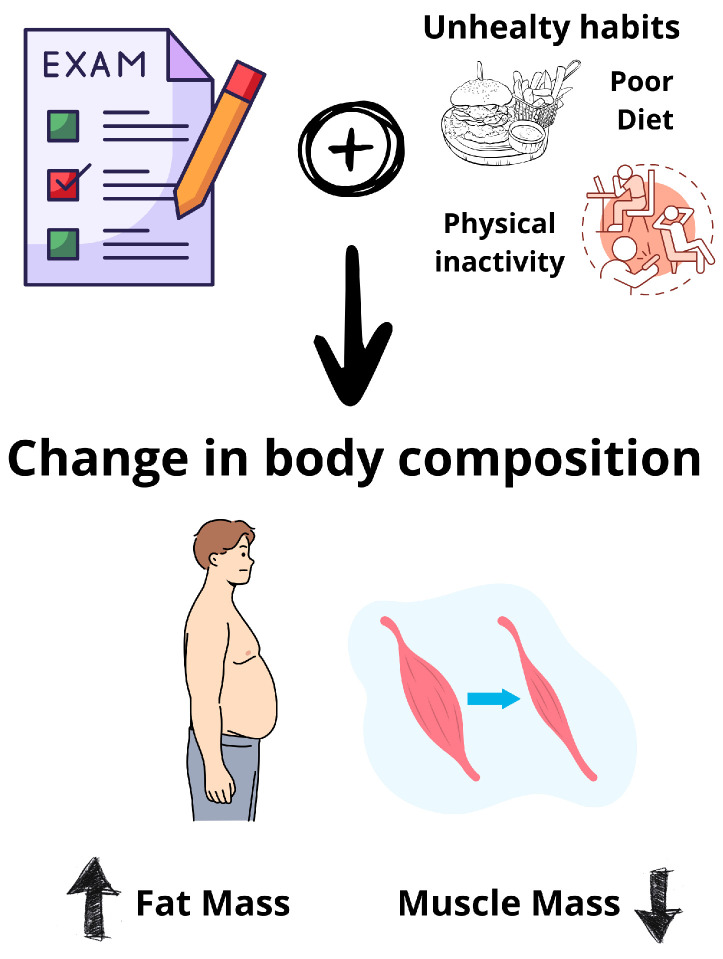
Conceptual summary of the impact of exam periods on student health. The infographic illustrates how academic stress leads to unhealthy habits, including poor diet and physical inactivity, which subsequently result in increased fat mass and decreased muscle mass, reflecting adverse changes in body composition.

Poor dietary habits and a sedentary lifestyle contribute to the deterioration of body composition. Among university students, both diet quality and physical activity levels decline significantly during exam preparation periods, leading to increased fat mass, decreased muscle mass, and elevated visceral fat. These findings underscore the importance of promoting healthy behaviors, particularly during periods of academic stress, to prevent long-term health risks (Figure 7).

## 6. Limitations

- Limited and homogeneous sample: All participants were second-year nursing students from a single institution (University Complutense of Madrid), which restricts the external validity and generalizability of the findings to other academic disciplines or university contexts.
- Participant attrition: Of the initial 142 students, only 72 completed both stages of the study. This loss may introduce selection bias, as students experiencing higher stress or more unhealthy behaviors may have been less likely to complete the follow-up.
- Lack of objective stress measurement: Although academic stress is hypothesized as a key factor influencing lifestyle changes, no validated tool (e.g., Perceived Stress Scale or Academic Stress Inventory) was used to assess it. This limits the ability to quantify its specific impact.
- Self-reported data: Both dietary habits (Kidmed) and physical activity (IPAQ) were assessed using self-administered questionnaires, which may be subject to social desirability bias and recall errors.
- Observational study design: The study’s correlational and observational nature precludes causal inference. Therefore, it cannot be concluded with certainty that exam preparation or stress directly caused the observed changes in body composition.
- Most participants were female (79.20%), which may limit the generalization of our findings to male university students. Future studies should aim to include more balanced gender representation to explore potential sex-specific trends in body composition changes during exam periods.

## 7. Future Research Directions

- Incorporate validated stress measurement tools: Future studies should assess perceived stress levels using validated scales (e.g., PSS), allowing for direct correlation between stress and changes in health-related behaviors and outcomes.
- Expand to other academic disciplines and institutions: Replicating the study across different fields of study and universities would enhance the generalizability and robustness of the findings.
- Intervention-based studies: Experimental or quasi-experimental designs could assess the effectiveness of specific interventions (e.g., brief educational sessions, mindfulness, or physical activity programs) implemented during exam periods to mitigate negative outcomes.
- Extended longitudinal follow-up: Investigating whether the changes in body composition are transient or sustained over time could provide insight into their potential long-term health implications.
- Objective measurement of diet and physical activity: The use of accelerometers, 24-h dietary recalls, or nutritional biomarkers could improve the accuracy and reliability of lifestyle data collection.
- The potential influence of the menstrual cycle on body composition measurements—particularly those obtained through bioimpedance—was not controlled for. Given that hormonal fluctuations across the menstrual cycle can affect hydration status and fat distribution, future studies should consider accounting for menstrual phase to improve the precision of body composition assessments in female participants.

## Figures and Tables

**Table 1:** Demographic and clinical variables in the initial data collection phase before exam preparation.

		n = 142
Age, mean (SD)		22.54 (7.38)
Gender, n (%)	Man	30.00 (21.10)
	Woman	112 (78.90)
Weight, mean (SD)		63.32 (10.79)
Height, mean (SD)		165.89 (7.94)
BMI, mean (SD)		23.04 (3.31)
Fat mass, mean (SD)		26.07 (8.37)
Body fat range, n (%)	Low	18.00 (12.70)
	Moderate	22.00 (15.50)
	High	42.00 (29.60)
	Very high	60.00 (42.30)
Muscular mass, mean (SD)		38.96 (5.37)
Body muscular range, n (%)	Low	28.00 (19.70)
	Moderate	72.00 (50.70)
	High	42.00 (29.60)
Smoking habit, n (%)	Yes	28.00 (19.70)
	No	114.00 (80.30)
Caffeine intake, n (%)	Yes	93.00 (65.50)
	No	49.00 (34.50)
Daily cups of coffee consumption, n (%)	Yes	1.59 (0.76)
Cola drink consumption, n (%)	Yes	38.00 (26.80)
	No	104.00 (73.20)
Energy drink consumption, n (%)	Yes	19.00 (13.40)
	No	123.00 (86.60)
Fruit or juice consumption, n (%)	Yes	118.00 (83.10)
	No	24.00 (16.90)
Intake of a second piece of fruit, n (%)	Yes	69.00 (48.60)
	No	73.00 (51.40)
Daily intake of fresh or cooked vegetables (once per day), n (%)	Yes	115.00 (81.00)
	No	27.00 (19.00)
Intake of fresh or cooked vegetables more than once daily, n (%)	Yes	73.00 (51.40)
	No	69.00 (48.60)
Intake of fish 2–3 times per week, n (%)	Yes	84.00 (59.20)
	No	58.00 (40.80)
Visits to fast food restaurants (≥1 time per week), n (%)	Yes	44.00 (31.00)
	No	98.00 (69.00)
Legume intake more than once weekly, n (%)	Yes	127.00 (89.40)
	No	15.00 (10.60)
Pasta or rice intake ≥ 5 times per week	Yes	55.00 (38.70)
	No	87.00 (61.30)
Cereal or cereal-derived product consumption at breakfast, n (%)	Yes	106.00 (74.60)
	No	36.00 (25.40)
Nut intake 2 to 3 times weekly, n (%)	Yes	81.00 (57.00)
	No	61.00 (43.00)
Olive oil consumption, n (%)	Yes	150.00 (98.60)
	No	2.00 (1.40)
Habitually skips breakfast, n (%)	Yes	19.00 (13.40)
	No	123.00 (86.60)
Breakfast includes a dairy product, n (%)	Yes	115.00 (81.00)
	No	27.00 (19.00)
Breakfast includes industrial pastries, n (%)	Yes	28.00 (19.70)
	No	114.00 (80.30)
Intake of at least 2 yogurts daily, n (%)	Yes	66.00 (46.50)
	No	76.00 (53.50)
Intake of sweets several times per day, n (%)	Yes	10.00 (7.00)
	No	132.00 (93.00)
Nutritional score, n (%)	Low quality	4.00 (2.80)
	Moderate quality	69.00 (48.60)
	High quality	69.00 (48.60)
Days of vigorous physical activity, mean (SD)		3.14 (1.40)
Minutes of vigorous physical activity, mean (SD)		253.49 (172.65)
Days of moderate physical activity, mean (SD)		2.91 (1.60)
Minutes of moderate physical activity, mean (SD)		189.79 (158.96)
At least 10 consecutive minutes of walking in the past 7 days, mean (SD)		6.32 (1.31)
Minutes of walking per day, mean (SD)		492 (404.31)
Daily sitting time, mean (SD)		394.51 (147.61)
Exercise score, n (%)	Low	7.00 (4.90)
	Moderate	21.00 (14.80)
	High	114.00 (80.30)

**Table 2:** Demographic and clinical variables in the second data collection phase, before and during exam preparation, with comparative analysis of results.

		Before (n = 72)	After (n = 72)	*p* (*)
Age, mean (SD)		21.94 (5.52)	21.94 (5.52)	
Gender, n (%)	Man	15.00 (20.80)	15.00 (20.80)	
	Woman	57.00 (79.20)	57.00 (79.20)	
Weight, mean (SD)		64.14 (10.52)	65.63 (11.96)	0.489
Height, mean (SD)		165.83 (8.30)	165.83 (8.30)	1
BMI, mean (SD)		23.32 (3.45)	24.10 (3.86)	0.355
Fat mass, mean (SD)		25.43 (8.20)	28.79 (8.35)	0.016
Body fat range, n (%)	Low	18.00 (12.70)	6.00 (8.30)	0.028
	Moderate	22.00 (15.50)	6.00 (8.30)	
	High	25.00 (29.60)	14.00 (19.40)	
	Very high	28.00 (38.90)	46.00 (63.90)	
Muscular mass, mean (SD)		38.96 (5.37)	39.70 (5.35)	
Body muscular range, n (%)	Low	11.00 (15.30)	35.00 (48.60)	<0.001
	Moderate	35.00 (48.60)	31.00 (43.10)	
	High	26.00 (36.10)	6.00 (8.30)	
Smoking habit, n (%)	Yes	11.00 (15.30)	11.00 (15.30)	1
	No	61.00 (84.70)	61.00 (84.70)	
Caffeine intake, n (%)	Yes	42.00 (58.30)	45.00 (63.40)	0.536
	No	30.00 (41.70)	26.00 (36.60)	
Daily cups of coffee consumption, n (%)	Yes	1.60 (0.82)	1.60 (0.82)	1
Cola drink consumption, n (%)	Yes	18.00 (25.00)	22 (30.60)	0.456
	No	54.00 (75.00)	50.00 (69.40)	
Energy drink consumption, n (%)	Yes	8.00 (11.10)	11.00 (15.30)	0.460
	No	64.00 (88.90)	61.00 (84.70)	
Fruit or juice consumption, n (%)	Yes	61.00 (84.70)	59.00 (81.90)	0.655
	No	11.00 (15.30)	13.00 (18.10)	
Intake of a second piece of fruit, n (%)	Yes	38.00 (52.80)	24.00 (33.30)	0.018
	No	34.00 (47.20)	48.00 (66.70)	
Daily intake of fresh or cooked vegetables (once per day), n (%)	Yes	57.00 (79.20)	49.00 (68.10)	0.130
	No	15.00 (20.80)	23.00 (31.90)	
Intake of fresh or cooked vegetables more than once daily, n (%)	Yes	31.00 (43.10)	31.00 (43.10)	1
	No	41.00 (56.90)	41.00 (56.90)	
Intake of fish 2–3 times per week, n (%)	Yes	38.00 (52.80)	35.00 (48.60)	0.617
	No	34.00 (47.20)	37.00 (51.40)	
Visits to fast food restaurants (≥1 time per week), n (%)	Yes	35.00 (48.60)	38.00 (52.80)	0.024
	No	37.00 (51.40)	34.00 (47.20)	
Legume intake more than once weekly, n (%)	Yes	61.00 (84.70)	57.00 (79.20)	0.386
	No	11.00 (15.30)	15.00 (20.80)	
Pasta or rice intake ≥ 5 times per week	Yes	29.00 (40.30)	23.00 (31.90)	0.298
	No	43.00 (59.70)	49.00 (68.10)	
Cereal or cereal-derived product consumption at breakfast, n (%)	Yes	52.00 (72.20)	53.00 (73.60)	0.851
	No	20.00 (27.80)	19.00 (26.40)	
Nut intake 2 to 3 times weekly, n (%)	Yes	39.00 (54.20)	39.00 (54.20)	1
	No	33.00 (45.80)	33.00 (45.80)	
Olive oil consumption, n (%)	Yes	71.00 (98.60)	68.00 (94.40)	0.172
	No	1.00 (1.40)	4.00 (5.60)	
Habitually skips breakfast, n (%)	Yes	10.00 (13.90)	37.00 (51.40)	<0.001
	No	62.00 (86.10)	35.00 (48.60)	
Breakfast includes a dairy product, n (%)	Yes	58.00 (80.60)	53.00 (73.60)	0.322
	No	14.00 (19.40)	19.00 (26.40)	
Breakfast includes industrial pastries, n (%)	Yes	17.00 (23.60)	33.00 (45.80)	0.005
	No	55.00 (76.40)	39.00 (54.20)	
Intake of at least 2 yogurts daily, n (%)	Yes	42.00 (58.30)	40.00 (55.60)	0.736
	No	30.00 (41.70)	32.00 (44.40)	
Intake of sweets several times per day, n (%)	Yes	5.00 (6.90)	21.00 (29.20)	0.001
	No	67.00 (93.10)	51.00 (70.80)	
Nutritional score, n (%)	Low quality	3.00 (4.20)	17.00 (24.30)	<0.001
	Moderate quality	36.00 (50.00)	39.00 (55.70)	
	High quality	33.00 (45.80)	14.00 (20.00)	
Overall dietary score, n (%)		7.55 (1.97)	5.45 (2.14)	<0.001
Days of vigorous physical activity, mean (SD)		3.10 (1.41)	0.72 (1.07)	<0.001
Minutes of vigorous physical activity, mean (SD)		229.02 (169.71)	30.69 (60.25)	<0.001
Days of moderate physical activity, mean (SD)		2.83 (1.63)	1.15 (1.64)	<0.001
Minutes of moderate physical activity, mean (SD)		196.17 (165.59)	57.50 (125.03)	<0.001
At least 10 consecutive minutes of walking in the past 7 days, mean (SD)		6.41 (1.11)	3.79 (2.23)	<0.001
Minutes of walking per day, mean (SD)		506.55 (391.85)	168.83 (174.90)	<0.001
Daily sitting time minutes, mean (SD)		408.24 (154.28)	543.61 (237.48)	<0.001
Exercise score, n (%)	Low	3.00 (4.20)	43.00 (59.70)	<0.001
	Moderate	8.00 (11.10)	21.00 (29.20)	
	High	61.00 (84.70)	8.00 (11.10)	

* *p*-values calculated using chi-square test for categorical variables.

**Table 3:** Relationship between diet quality and body composition.

	Fat	Visceral Tissue	Muscle
Low-quality diet (SD)	30.10 (8.73)	4.14 (2.51)	35.16 (5.41)
Moderate-quality diet (SD)	29.60 (7.35)	3.57 (2.45)	35.71 (3.94)
High-quality diet (SD)	24.91 (10.29)	2.78 (1.96)	38.64 (5.16)

**Table 4:** Relationship between physical activity and body composition.

	Fat	Visceral Tissue	Muscle
Low physical activity (SD)	30.31 (7.72)	3.90 (2.67)	35.25 (4.51)
Moderate physical activity (SD)	26.40 (8.34)	3.04 (1.77)	37.81 (4.15)
Vigorous physical activity (SD)	26.95 (10.73)	2.75 (1.58)	37.33 (5.52)

## Data Availability

The original contributions presented in this study are included in the article. Further inquiries can be directed to the corresponding author.

## Abbreviations

The following abbreviations are used in this manuscript:

BMI	Body mass index
IPAQ	International Physical Activity Questionnaire
SD	Standard deviation
T2D	Type 2 diabetes

## Appendix A

**Table A1:** Modified Kidmed questionnaire: Adherence to the mediterranean diet in childhood.

Item	Adherence to the Mediterranean Diet	Points
1	I eat a dairy product for breakfast (milk, yogurt, etc.)	+1
2	I eat a cereal or cereal-based product for breakfast (e.g., bread)	+1
3	I skip breakfast	−1
4	I eat industrial pastries, cookies, or cakes for breakfast	−1
5	I consume one fruit or a natural juice every day	+1
6	I consume a second piece of fruit every day	+1
7	I eat fresh (e.g., salad) or cooked vegetables at least once a day	+1
8	I eat fresh or cooked vegetables more than once a day	+1
9	I eat 2 yogurts and/or 40 g of cheese daily	+1
10	I eat fish at least 2–3 times a week	+1
11	I go to a fast-food restaurant (e.g., burger place, pizzeria) or order food for delivery at least once a week	−1
12	I like legumes (chickpeas, lentils, etc.) and eat them 2–3 times a week	+1
13	I eat pasta or rice almost daily (5 or more times a week)	+1
14	Olive oil is used at home	+1
15	I frequently eat unsalted nuts (at least 2–3 times a week)	+1
16	I eat sweets, candies, or ice cream several times a day	−1
17	I snack daily between meals (e.g., chips, salty snacks, popcorn)	−1
18	I drink sugary beverages, packaged juices, and/or milkshakes every day	−1
19	I drink three or more glasses of water per day	+1

**Table A2:** Physical activity classification based on IPAQ—Short Form.

Level	Criteria
High	- Vigorous-intensity activity on at least 3 days, achieving a minimum of 1500 MET-min/week, or - 7 or more days of any combination of walking, moderate- or vigorous-intensity activities achieving a minimum of 3000 MET-min/week
Moderate	- 3 or more days of vigorous-intensity activity of at least 20 min/day, or - 5 or more days of moderate-intensity activity and/or walking of at least 30 min/day, or - 5 or more days of any combination of walking, moderate- or vigorous-intensity activities achieving a minimum of 600 MET-min/week
Low	- Does not meet criteria for high or moderate physical activity levels

**Table A3:** Body fat classification by age and sex (percentages).

Age (Years)	Low	Normal	High	Very High
Men				
10–14	<11%	11–16%	16.1–21%	>21%
15–19	<12%	12–17%	17.1–22%	>22%
20–29	<13%	13–18%	18.1–23%	>23%
30–39	<14%	14–19%	19.1–24%	>24%
40–49	<15%	15–20%	20.1–25%	>25%
50–59	<16%	16–21%	21.1–26%	>26%
60–69	<17%	17–22%	22.1–27%	>27%
70+	<18%	18–23%	23.1–28%	>28%
Women				
10–14	<16%	16–21%	21.1–26%	>26%
15–19	<17%	17–22%	22.1–27%	>27%
20–29	<18%	18–23%	23.1–28%	>28%
30–39	<19%	19–24%	24.1–29%	>29%
40–49	<20%	20–25%	25.1–30%	>30%
50–59	<21%	21–26%	26.1–31%	>31%
60–69	<22%	22–27%	27.1–32%	>32%
70+	<23%	23–28%	28.1–33%	>33%

**Table A4:** Muscle mass classification by age and sex (kg).

Age (Years)	Low	Normal	High
Men			
10–14	<44	44–57	>57
15–19	<43	43–56	>56
20–29	<42	42–54	>54
30–39	<41	41–52	>52
40–49	<40	40–50	>50
50–59	<39	39–48	>48
60–69	<38	38–47	>47
70+	<37	37–46	>46
Women			
10–14	<36	36–43	>43
15–19	<35	35–41	>41
20–29	<34	34–39	>39
30–39	<33	33–38	>38
40–49	<31	31–36	>36
50–59	<29	29–34	>34
60–69	<28	28–33	>33
70+	<27	27–32	>32
